# Consumption—Its Relation to Man and His Civilization—Its Prevention and Cure

**Published:** 1906-08

**Authors:** 


### BOOK REVIEWS.

CONSUMPTION—ITS RELATION TO MAN AND HIS CIVILIZATION—ITS PREVENTION AND CURE. By John Bessner Huber, A.M., M.D. Fellow of the New York Academy of Medicine; Member of the National Association for the Study and Prevention of Tuberculosis; Visiting Physician to St. Joseph's Hospital for Consumptives; Member of the Advisory Board, the New Mexico Cottage Sanatorium, etc. J. B. Lippincott Co., Publishers, Philadelphia and London.

The title denotes the scope of this work, being an able, "comprehensive exposition of the effect which consumption has had upon civilization and a consideration of its relation to human affairs." It deals with problems equally as important as the purely medical aspect of this disease, i. e., its connection with economics, legislation, sociology, etc., and is not only a valuable book for physicians, but could with much profit be read by educated laymen, and especially those with tuberculosis.

The chapters are short and devoted to a single subject, which is written in a clear, interesting style, and from the first one realizes that the author is thoroughly conversant with the subject in its broadest sense.

Regarding human and bovine tuberculosis, Dr. Huber does not agree with Koch, but believes they are identical. The predisposition, prevention and cure are satisfactorily discussed, and much that is of importance is given about tents, dispensaries, hospitals, sanatoria, etc. A thorough diffusion of the knowledge of the relation of tuberculosis to over-crowding occupations, habits, social customs, the house, poverty, alcoholism, and many preventable conditions he so clearly presents, if generally known, would, within a few years, greatly lessen the harvest of the relentless "white plague." We commend this book unreservedly to physicians and laymen.

